# Evaluation of age-specific causes of death in the context of the Italian longevity transition

**DOI:** 10.1038/s41598-022-26907-3

**Published:** 2022-12-31

**Authors:** Andrea Nigri, José Manuel Aburto, Ugofilippo Basellini, Marco Bonetti

**Affiliations:** 1 Department of Economics, Management and Territory, University of Foggia, Foggia, Italy; 2 Department of Social and Political Sciences, Bocconi University, Milan, Italy; 3 Leverhulme Centre for Demographic Science, Nuffield College, University of Oxford, Oxford, UK; 4 Interdisciplinary Centre on Population Dynamics, University of Southern Denmark, Odense, Denmark; 5 Department of Population Health, London School of Hygiene and Tropical Medicine, London, UK; 6 Max Planck Institute for Demographic Research (MPIDR), Rostock, Germany; 7 Carlo F. Dondena Research Centre, Bocconi University, Milan, Italy

**Keywords:** Health care, Statistics

## Abstract

In many low-mortality countries, life expectancy at birth increased steadily over the last century. In particular, both Italian females and males benefited from faster improvements in mortality compared to other high-income countries, especially from the 1960s, leading to an exceptional increase in life expectancy. However, Italy has not become the leader in longevity. Here, we investigate life expectancy trends in Italy during the period 1960–2015 for both sexes. Additionally, we contribute to the existing literature by complementing life expectancy with an indicator of dispersion in ages at death, also known as lifespan inequality. Lifespan inequality underlies heterogeneity over age in populating health improvements and is a marker of uncertainty in the timing of death. We further quantify the contributions of different age groups and causes of death to recent trends in life expectancy and lifespan inequality. Our findings highlight the contributions of cardiovascular diseases and neoplasms to the recent increase in life expectancy but not necessarily to the decrease in lifespan inequality. Our results also uncover a more recent challenge across Italy: worsening mortality from infectious diseases and mortality at older age.

## Introduction

Life expectancy has increased considerably across the world over the last century. In high-income countries, past increases were mostly attributed to declines in infant and child mortality, and more recently to mortality reductions at older ages^1,2^. Despite the recent life expectancy deceleration in developed countries^3,4^, in 2019, Italy stood as a country with one of the highest life expectancy in Europe (second only to Spain in the European Union (EU), and 2 years more than the EU average) and globally. Since 1900, female life expectancy at birth has increased from 50 to 84.7 years, by an average of 2.9 years per decade^5^. Progress in reducing mortality since the beginning of the 1960s is at the heart of the Italian longevity improvement. A combination of a proficient health system and healthy lifestyles have contributed to this progress. For example, in 1978, the Italian government established the Italian National Health Service (NHS). The NHS may be considered as one of the best practices among national health frameworks worldwide, ensuring universal coverage. Surgeries and medical treatment are provided for all citizens, regardless of their income. Moreover, prescription drugs, specialist visits, and diagnostic tests are freely provided for people suffering from chronic diseases, older people (aged> 65 years), people in the low income group and pregnant women.

However, public health financing and spending have been reduced and put under pressure in the most recent years, resulting in a decrease in the share of gross domestic product (GDP) directed to health expenditures, and in 2015, the per capita expenditure was below the EU average^6^. As such, an aging population and the recent decrease in public health financing might pose several challenges to the future of Italian longevity^7^. Hence, it is imperative to study Italian mortality in this context and analyze recent trends in life expectancy. Because of the recent changes in health expenditure, it is possible that mortality from some causes of death may have worsened in recent years, holding back life expectancy improvements. Nevertheless, the current literature does not provide any contribution that accurately investigates this issue.

While life expectancy is an important indicator of longevity, it conceals variation in lifespans or lifespan inequality, which can be substantial^8^. Lifespan inequality matters because it reflects individuals’ uncertainty in the timing of death, which can be important for life course decisions^9^, and it measures how uneven improvements in mortality are at the population level^10^. From a public health perspective, greater lifespan inequality implies increasing vulnerability at the societal level and the consequent ineffectiveness of policies to protect individuals against life course risks^11^. Hence, analyzing both life expectancy and lifespan inequality in the context of mortality developments provides a more comprehensive overview of longevity changes. Previous evidence showed that Italian life expectancy has improved, accompanied by reductions in lifespan inequality^12^. However, there is still a lack of evidence on the contributions of different age groups and causes of death to recent mortality changes in Italy.

In Italy, the analysis of leading causes of death shows that cardiovascular diseases accounted for 30% of all deaths in 2012, followed by malignant neoplasms of the trachea, bronchus, and lung^13^. Transportation-related accidents were found to be leading in the age group of 15–24 years, especially among males. The number of deaths in the age group 65–84 years accounted for about 50% of overall mortality. Here, the top leading causes were ischemic heart diseases and cerebrovascular diseases, respectively, for males and females. At older ages (85 years and above), the most frequent causes were the diseases of the circulatory system, especially heart diseases^13^.

In this article, we provide two main contributions. First, we analyze patterns of Italian mortality since the 1960s through life expectancy complemented with lifespan inequality. Most literature in this area focuses on average indicators of mortality, such as age-standardized mortality rates or life expectancy. Our work highlights the role of lifespan inequality as an additional indicator of dispersion in ages at death. We describe the observed changes in both indicators since 1960 by sex. A second contribution is that we quantify how mortality in different age groups and from eight groups of causes of death contributed to changes in life expectancy and lifespan inequality. Our analysis underscores how improvements in cardiovascular mortality and deaths due to cancer have contributed to increasing life expectancy. While these two groups of causes of death have been identified as the main drivers of life expectancy improvements, little is known about how they impact lifespan inequality trends. Moreover, we shed light on how they have offset gains in life expectancy in the last decades. This framework allows us to thoroughly analyze mortality changes and to determine the contributions of different causes of death to the observed changes.

## Results

### Overall lonegevity

**Figure 1:**
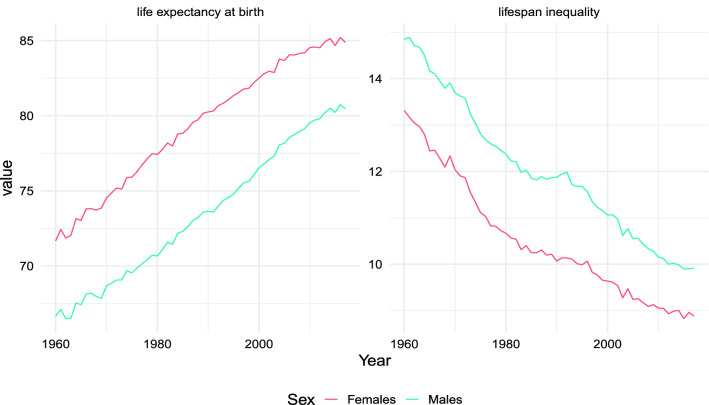
Lifespan inequality and life expectancy trends from 1960 to 2015 by sex. *Source*: authors’ own elaborations on data from the HMD^5^.

Females experienced a decrease in lifespan inequality and a steady increase in life expectancy throughout the period 1960–2015, from 71.7 years to 84.7 years (see Fig. 1). For males, the increase in life expectancy was sustained throughout the period, increasing from 66.7 to 80.2 years, while the decrease in lifespan inequality did not occur in the period 1983–1992, followed by resumption of the decreasing trend thereafter. Notably, during such stagnation in inequality no change in the increasing trend in life expectancy was observed.

### CoD analysis

**Figure 2:**
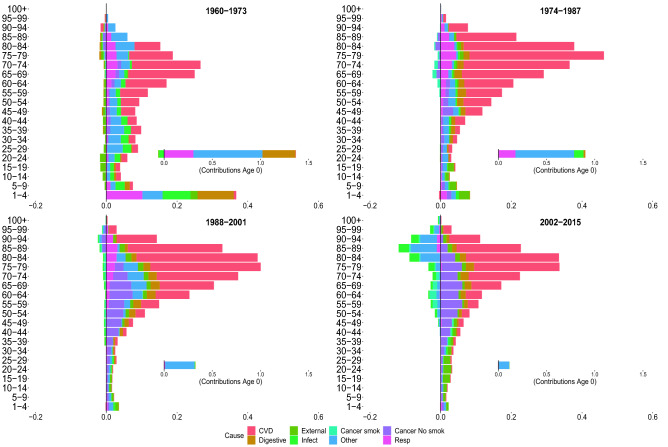
Age and cause contributions to changes in life expectancy for Italian females.

**Figure 3:**
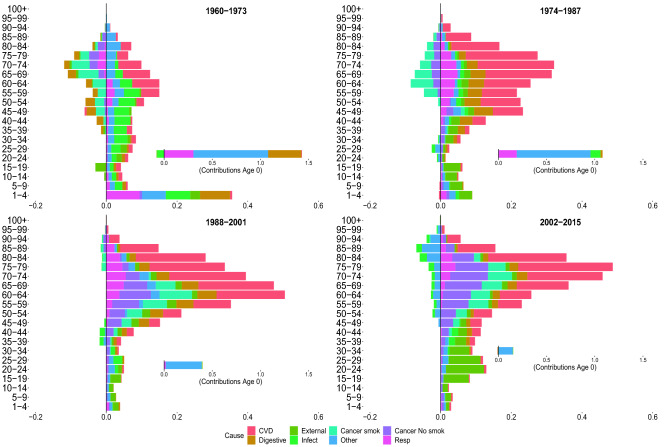
Age and cause contributions to changes in life expectancy for Italian males.

**Figure 4:**
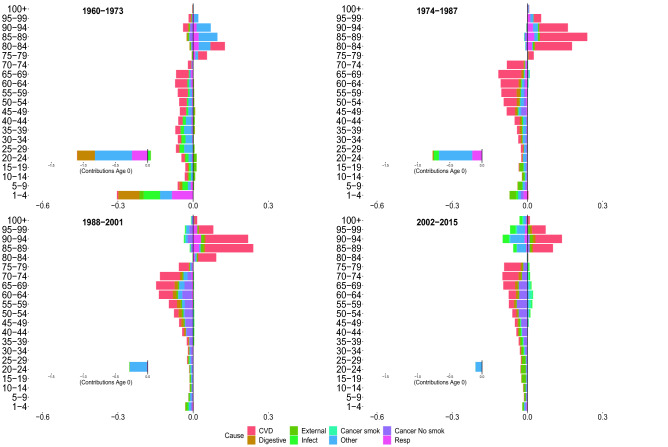
Age and cause contributions to changes in lifespan inequality for Italian females.

**Figure 5:**
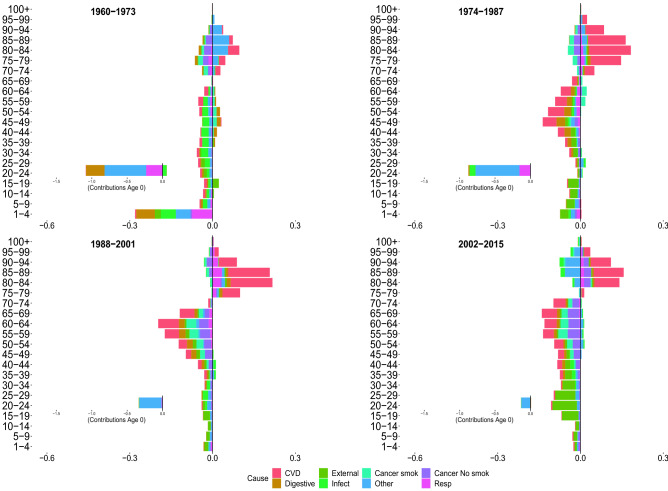
Age and cause contributions to changes in lifespan inequality for Italian males.

Figures 2, 3, 4 and 5 show the contributions of the various age groups and causes of death to changes in life expectancy (*e*(0)) and lifespan inequality ($e^\dagger$) from 1960 to 2015, focusing on four periods of 13 years each (1960–1973, 1974–01987, 1988–2001 and 2002–2015). The inner graph shows the contribution of improvements in infant mortality (which were substantial, and therefore are shown on a different scale). The cause-specific contributions for all age–groups are shown in Tables 1 and 2.

###  1960–1973

Between 1960–1973, Italian life expectancy increased from 71.7 to 75.1 years for females and 66.7 to 69.1 years for males. For both sexes, the improvement in life expectancy was mainly due to reductions in digestive, respiratory and mortality at very young ages (0 to 5 years).

For females, mid- and old-age groups contributed substantially to increasing life expectancy due to improvements from cardiovascular diseases. For males, in the same age groups, longevity increased due to a reduction in infectious and cardiovascular mortality, with the latter being more moderate than for females. Both females and males experienced a slight increase in infectious diseases at birth. Cancer attributable to smoking contributed negatively for adult and old ages in males. For both sexes, lifespan inequality was reduced because of improvements in mortality during the first years of life (especially at age 0).

###  1974–1987

Between 1974 and 1987, female and male life expectancy increase, mostly due to reductions in cardiovascular mortality from age 45 onward. Moreover, there was a smaller but continued reduction in infectious, respiratory and other causes in infancy; mid- and old-age smoking-related mortality reduced life expectancy for males, while this negative contribution was practically absent in females. Peculiar patterns emerged for lifespan inequality, which continued to decline for both sexes but displayed a short-term stagnation between 1983 and 1992 for males. As we noted above, this pattern did not coincide with stagnation in life expectancy. While changes in life expectancy were fueled by positive increases at all ages, lifespan inequality reductions are mainly attributable to infant mortality and were partially offset by positive contributions by CVD at older ages. Finally, for both sexes and measures, we observed that digestive diseases ended the positive role that they played in the first period.

### 1988–2001

In the third period (between 1988 and 2001), Italian life expectancy increased from 79.7 to 82.8 and 73.2 to 76.8 year, for females and males, respectively. For both sexes and measures, the first relevant effects of cancer emerged, providing a positive contribution. Prior to 1988–2001, there was no significant evidence of the contribution of cancer (other than smoking-related cancers) on improvements in longevity. In this period, the positive cancer contribution occurred in the mid- and adult-ages between 30–75 years. For males, the short stagnation of lifespan inequality until 1992 was followed by continued reductions. This is shown by both negative and positive contributions in lifespan inequality changes that are mainly attributable to cardiovascular and cancer mortality at different ages. This pattern is not expressed in the life expectancy that exhibits mostly positive contributions. We also note the decreasing impact played by mortality at infant ages as compared to previous periods.

### 2002–2015

Between 2002 and 2015, life expectancy increased by approximately 1.7 years for females and 3.2 years for males. For both sexes, improvements are driven by the reduction in mortality from cardiovascular disease in old age. Specifically, the population aged 80 and over contributes 30% for males, and almost 50% for females, to the total reduction in mortality from CVD diseases. The significance of cardiovascular diseases among males should be emphasized, which in tandem with smoking and non-smoking cancers, provide the greatest contributions in mid- and old-age improvements in life expectancy. Another interesting pattern that emerged in the adult ages is the constant, albeit modest, positive contribution of external causes, and negative contribution (especially for females) of infectious and other causes. Age-specific contributions from cancer reduction differ between the last two periods. Between 2002 and 2015, the positive contributions start from ages 35–40 up to 80–85. In contrast, in the previous period, the age groups involved are 30–35 years to 70–75. While life expectancy shows mainly positive contributions, lifespan inequality, on the other hand, shows both positive and negative contributions. This may be the result of a slight deceleration in the downtrend over the last five years of the analysis for both sexes. We underline for both sexes the negative contributions of the elderly, which are mainly attributable to cardiovascular mortality. There are noteworthy sex differences in external mortality. Males recorded more than twice the cumulative gain in *e*(0) from external-related mortality than females, with a reduction in lifespan inequality that is four times more than that experienced by females.

**Table 1:** Life expectancy absolute and percentage contributions aggregated over ages and specific by causes of deaths.

Sex	Cause of death	1960–1973	1974–1987	1988–2001	2002–2015
Females	CVD	1 (29.09%)	1.88 (51.51%)	1.65 (53.92%)	1.17 (68.82%)
	Digestive	0.45 (13.09%)	0.19 (5.21%)	0.199 (6.21%)	0.15 (8.82%)
	External	$-0.1$ (−2.91%)	0.13 (3.56%)	0.15 (4.90%)	0.19 (11.18%)
	Infectious	0.24 (6.98%)	0.15 (4.11%)	−0.01 (−0.33%)	−0.12 (−7.06%)
	Cancer smoking	−0.02 (−0.58%)	−0.04 (−1.10%)	−0.04 (−1.30%)	−0.06 (−3.53%)
	Other	1.24 (36.07%)	0.84 (23.01%)	0.55 (17.97%)	−0.11 (−6.47%)
	Cancer no smoking	0.038 (1.10%)	0.08 (2.19%)	0.39 (12.75%)	0.50 (29.41%)
	Respiratory	0.41 (17.16%)	0.42 (11.51%)	0.18 (5.88%)	−0.02 (−1.18%)
Males	CVD	0.45 (18.67%)	1.34 (40.36%)	1.40 (39.00%)	1.30 (41.41%)
	Digestive	0.30 (12.45%)	0.38 (11.45%)	0.38 (10.57%)	0.19 (6.05%)
	External	0.01 (0.41%)	0.33 (9.94%)	0.24 (6.69%)	0.52 (16.56%)
	Infectious	0.4 (16.60%)	0.18 (5.42%)	0.01 (0.28%)	−0.06 (−1.91%)
	Cancer smoking	−0.26 (−10.79%)	−0.23 (−6.93%)	0.28 (7.80%)	0.36 (11.46%)
	Other	1.17 (48.55%)	0.90 (27.11%)	0.55 (15.32%)	−0.03 (−0.96%)
	Cancer no smoking	−0.10 (−4.15%)	−0.06 (−1.81%)	0.42 (11.70%)	0.70 (22.29%)
	Respiratory	0.44 (18.26%)	0.48 (14.46%)	0.31 (8.64%)	0.16 (5.10%)

**Table 2:** Lifespan inequality absolute and percentage contributions aggregated over ages and specific by causes of deaths.

Sex	Cause of death	1960–1973	1974–1987	1988–2001	2002–2015
Females	CVD	−0.26 (14.69%)	0.08 (−7.77%)	0.17 (−27.87%)	−0.03 (4.17%)
	Digestive	−0.40 (22.60%)	−0.10 (9.71%)	−0.08 (13.11%)	−0.04 (5.56%)
	External	0.04 (−2.26%)	−0.06 (5.83%)	−0.03 (4.92%)	−0.09 (12.50%)
	Infectious	−0.16 (9.04%)	−0.11 (10.68%)	−0.01 (1.64%)	−0.06 (8.33%)
	Cancer smoking	0.00 (0%)	0.01 (-0.97%)	0.00 (0.00%)	0.01 (−1.39%)
	Other	−0.61 (34.46%)	−0.61 (59.22%)	−0.39 (63.93%)	−0.22 (30.56%)
	Cancer no smoking	−0.08 (4.52%)	−0.1 (9.71%)	−0.30 (49.19%)	−0.25 (34.72%)
	Respiratory	−0.30 (16.95%)	−0.14 (13.59%)	0.03 (−4.92%)	−0.04 (5.56%)
Males	CVD	−0.01 (0.62%)	0.26 (−22.61%)	0.18 (−23.68%)	0.01 (−0.92%)
	Digestive	−0.34 (20.99%)	−0.15 (13.04%)	−0.09 (11.84%)	−0.06 (5.56%)
	External	−0.04 (2.47%)	−0.20 (17.39%)	−0.12 (15.79%)	−0.37 (34.26%)
	Infectious	−0.20 (12.35%)	−0.09 (7.83%)	−0.03 (3.95%)	−0.06 (5.56%)
	Cancer smoking	0.00 (0%)	−0.04 (3.48%)	−0.17 (22.37%)	−0.15 (13.89%)
	Other	−0.56 (34.57%)	−0.62 (53.91%)	−0.35 (46.05%)	−0.29 (26.85%)
	Cancer no smoking	−0.09 (5.56%)	−0.12 (10.43%)	−0.22 (28.95%)	−0.18 (16.67%)
	Respiratory	−0.38 (23.46%)	−0.19 (16.52%)	0.04 (−5.25%)	0.02 (−1.85%)

## Discussion

We studied how mortality in different age groups and broad causes of death contributes to changes in longevity and lifespan inequality. To assess these differences, we compared the Italian mortality with the patterns observed in different periods, identified with consecutive time windows of 13 years each, from 1960 to 2015.

Through our analysis, it is possible to gain a better understanding of the Italian longevity transition by looking at the patterns of life expectancy alongside lifespan inequality. Italian life expectancy has increased considerably for both sexes compared to the life expectancy evolution in other countries around the world. This positive trend has been accompanied with substantial reductions in lifespan inequality. This means that Italians are not only living longer but also their lifespans have become more predictable, which can have implications for life course decisions, such as when to invest in education or when to retire^9,14^. From a public health perspective, this finding reflects that improvements in mortality have become more egalitarian over the age span. Even though Italy has experienced these improvements, the country has not been the record holder of life expectancy^5,15^. Nevertheless, Italy stands as one of the main performers in achieving high levels of life expectancy and low lifespan inequality. This is consistent with previous research highlighting the negative association between life expectancy and lifespan inequality^10,16–19^. Life expectancy record holders tend to have the lowest lifespan inequality, which underscores the potential for improvement in both indicators in the case of Italy.

Consistently with previous evidence, our results show that cardiovascular and cancer mortality contributed substantially to increasing life expectancy (see Tables 1 and 2), but they do not account for most of the declines in lifespan inequality^20^. Hence, in these causes of death, there are no consistent relationships between changes in life expectancy and lifespan inequality. Causes of death that occur at younger ages, such as lifestyle-related causes (e.g., digestive and external diseases), played an increasingly important role over time in life expectancy and lifespan inequality improvements. We document a short period of stagnation in lifespan inequality for males in 1983–1992. Evidence suggests that recent periods of stagnation are generally driven by stalls in mortality improvements in working young-adult ages (see, e.g., Aburto & van Raalte (2018)^21^), especially in individuals pertaining to lower socioeconomic status groups^8^. More studies looking at subgroups in Italy are needed to further understand the observed national trends in mortality improvements.

Recent studies in high income countries have primarily focused on the impact of cardiovascular and cancer mortality on life expectancy improvements. Although these causes are crucial in targeting public resource intervention, here, we aim to shed light on peculiar features observed in the Italian case and often not analyzed in other studies: the cases of digestive and infectious diseases. In our analysis, despite the sharp increase in overall longevity, especially in the first period, we note unexpected negative contributions due to infectious diseases (and a similar contribution from respiratory diseases) for both sexes. This insight led us to deepen the analysis by focusing on the yearly variations, in turn, allowing us to underline the important contribution of respiratory and infectious causes in the period 1967–1968 (see Figures A.3 and A.4 in the Appendix). Although identifying causal relationships is out of the scope of this paper, since we do not have sufficient evidence, this result is possibly not coincidental, and we try to offer some speculative views. Aiming at uncovering the presumable nature of this reduction, we found potential evidence in the spread of the Hong Kong flu, also known as the 1968 flu pandemic. We consider a possible hypothesis to explain the unexpected longevity reduction due to infectious and respiratory diseases, finding potential evidence in the widespread outbreak of acute respiratory disease. The 1968 pandemic was caused by an influenza A (H3N2) virus, the so-called Hong Kong influenza. It was first noted in the United States in September 1968. According to the CDC, the estimated number of deaths was 1 million worldwide and about 100,000 in the United States^22,23^. According to reports from correspondents of The New York Times, the influenza virus also struck Europe during the winter. In Italy, the “ISS,” or Italian National Institute of Health, claims that the excess mortality attributable to pneumonia and flu associated with this pandemic was estimated at 20,000 deaths. Further evidence, information or specific data may allow one to better understand the issue.

A further contribution to the observed changes in mortality comes from digestive diseases, which positively contribute to the longevity increase in 1967. Such evidence is also partly supported by previous studies^24^. The disappearance of mortality attributable to digestive causes could be partly linked to the theory of the epidemiological revolution. In particular, it could be related to improvements in lifestyles due to greater food awareness among the population. Vercelli et al.^25^ notes that over the same period, there was a significant association between increasing calorie intake over time and falling population mortality from a class of causes, including digestive. Recently, some studies in Italy^26^, using individual-level data, have investigated the reduction of digestive diseases, highlighting the component between weight change and mortality attributable to digestive causes.

## Conclusions

Looking at the overall Italian longevity levels, our results confirm the presence of a negative correlation between life expectancy and lifespan inequality throughout the period 1960–2015. Our findings suggest that the Italian public health policy may foster future gains in life expectancy by targeting the reduction of cancer and CVD diseases for both sexes and external causes mortality for men. The reduction of cardiovascular mortality provides the greatest contributions to mid- and old-age improvements in life expectancy. Importantly, causes of death that increase life expectancy may not necessarily also reduce lifespan inequality. Indeed, improvements in mortality always increase life expectancy, but if these improvements occur at older ages, lifespan inequality may increase too. Our study offers evidence of this phenomenon, underling the positive contribution to life expectancy due to a reduction in CVD and cancer, that has not been translated into a reduction in inequality.

Causes of death have become increasingly diverse in recent decades as a result of gains in life expectancy and improvement in early diagnoses, with a consequent challenge to healthcare systems^27^. Thus, monitoring inequality in longevity and how it changes over time is of prime importance to understand mechanisms of mortality improvement and adopting policies that enhance health equity.

In conclusion, the systematic evolution of life expectancy and lifespan inequality is crucial for policymakers and health system experts, in order to adopt targeted interventions for specific classes of disease. Our study contributes to this strand of research by highlighting mortality developments in the Italian context between 1960 and 2015.

## Methods

### Mortality indicators and data

Consider a non-negative random variable *X* that represents the duration between the time of birth and the time of death. This is called “lifetime” with cumulative distribution function $F(x)=P(X \le x)$, and survival function $S(x)=\mathbb {P}\left( X \ge x\right)$. This is the probability for an individual to survive until age *x*. The distribution of *X* is often expressed in terms of the force of mortality $\mu (x)$, defined as the instantaneous rate of death at age *x*, conditionally on surviving to that age:

1 $$\begin{aligned} \mu (x)=\lim _{\Delta x \rightarrow 0} \frac{\mathbb {P}(X \in [x, x+\Delta x) | X \ge x)}{\Delta x}. \end{aligned}$$

Note that the product $\mu (x) \Delta x$ can be interpreted as the approximate probability that an individual of age *x* dies in the $[x, x+\Delta x)$ time interval, given that she survived until *x*.

Period life expectancy at birth is the most widely used indicator of health and longevity^28^. It refers to the expected average age at death for a synthetic cohort of newborns, who experience the mortality risks of that time throughout their lifespan. Following standard demographic methods^29^, we define the residual life expectancy of those still alive at age *x* and calendar time *t* in a given population, as follows:

2 $$\begin{aligned} e(x,t)= \frac{\int _{x}^{\infty }S(y,t) dy}{S(x,t)}. \end{aligned}$$

Note that being alive at age *x* and calendar time *t* is equal to being born at $t-x$ and alive at age *x*.

While the concept of life expectancy is well-known, it conceals variation in ages at death or lifespan inequality. Lifespan inequality is less used in mortality studies. However, the case for using it alongside life expectancy has been recently argued^8^. There are different indicators to measure lifespan inequality, including the standard deviation or the Gini coefficient of the age-at-death distribution. These indicators are highly correlated between each other^19,30^. We decided to use the average years of life lost due to death, denoted by $e^\dagger (x,t)$, as defined by Vaupel and Canudas-Romo (2003)^31^. Lifespan inequality at age *x* and time *t* is defined as follows:

3 $$\begin{aligned} e^\dagger (x,t)= \frac{\int _{x}^{\infty } e(a,t) \cdot d(a,t) da}{S(x,t)}. \end{aligned}$$

This is an indicator that has a meaningful public health interpretation and a link to the lifetable entropy^32^.

### Data and classification of causes of death

We use period life tables by sex and single year of age (0–100+) from the Human Mortality Database^5^. We extracted death counts by cause of death from the WHO^33^, for both females and males, thus computing the proportion of deaths by cause, age, and sex in a given year,from 1960 to 2015, the last year available in the WHO.

The data in their original form were classified using the seventh, eighth, ninth, and tenth revisions of the International Classification of Diseases (ICD). For our purpose of capturing conditions that might have affected Italian longevity, we group deaths into eight major categories up to age 100+ (see Table 3). We consider smoking-related cancers to capture particular lifestyle behaviors. We also consider other cancers and cardiovascular diseases (CVD) as relevant causes of death capturing a great share of overall deaths. Furthermore, we use external mortality and respiratory, infectious, digestive, and the rest of the causes were classified as “other”. For ICD codes and details on the classification system, see Table 3.

**Table 3:** ICD codes and our classification into eight major groups.

Cause	ICD7	ICD8	ICD9	ICD10
Infectious	A001–A043, A104, A132, B001–B017, B043	A001–A044	B01–B07	A00–B99
Cancer Smoking	A050	A051	B101	C33–C34
Cancer no smoking	A044–A059, B018–B019	A045–A050, A052–A060	B08–B09, B100, B109, B11–B17	C except C33–C34
CVD	A070, A079–A086, B022, B024–B029	A080–A088	B25–B30	I00–I99
Respiratory	A087–A097, B030–B032	A089–A096	B31–B32	J00–98
Digestive	A098–A107, B033–B037	A097–A104	B33–B34	K00–K93
External	A138–A150, B047–B050	A138–A150	B47–B56	V00–Y89
Other	A137, B045, A060–A069, B020, A071–A078, A108–A137	A105–A137, B045, A061–A079	B46, B18–B24, B35–B46	R00–R99, D00–D48, D50–D89,E00–E88, F01–F99,G00–G98, H00–H57,H60–H93, K00–K92, L00–L98, M00–M99,N00–N98, O00–O99,P00–P96, Q00–Q99, R00–R99

Source: authors’ own elaborations on data from the WHO^33^.

Since cause-of-death data are available in 5-year age groups, we employ the Penalized Composite Link Model (PCLM)^34,35^ to ungroup data into single years of age. The method is based on the composite link model^36^, with a penalty added to ensure the smoothness of the target distribution^37^. Figures A.1 and A.2 in the Appendix, present the age pattern of the cause-specific mortality rates on the log scale, showing the consistency and reliability of our ICD classification.

### Decomposition methods

The decomposition of the changes in life expectancy and lifespan inequality by sex according to cause of death and age was performed for Italy from 1960 to 2015 using the linear integral model^38^. These decompositions allow us to attribute the ages and causes responsible for changes in life expectancy or lifespan inequality between any two periods, for instance between 2000 and 2015.

This method relies on the assumption that the dependent variable (e.g. life expectancy) is a differentiable function of the covariates (age-cause specific death rates) and that their effects are additive. It also leverages the assumption that covariates change gradually along a dimension (e.g. time), therefore, it is appropriate for decomposing time trends.

Suppose that *f* is a differentiable function of *n* covariates (e.g., each age-cause specific mortality rate) denoted by the vector $\varvec{A}=\left[ x_{1}, x_{2}, \ldots , x_{n}\right] ^{'}$. Assume that *f* and $\varvec{A}$ depend on the underlying dimension *t*, which is time, and that we have observations available at two time points $t_{1}$ and $t_{2}$. Assuming that $\varvec{A}$ is a differentiable function of *t* between $t_{1}$ and $t_{2}$, the difference in *f* between $t_{1}$ and $t_{2}$ can be expressed as follows^38^:

$$f_{2}-f_{1}=\sum _{i=1}^{n} \int _{x_{i}\left( t_{1}\right) }^{x_{i}\left( t_{2}\right) } \frac{\partial f}{\partial x_{i}} d x_{i}=\sum _{i=1}^{n} c_{i},$$

where $c_{i}$ is the total change in *f* produced by changes in the *i* th covariate, $x_{i}$.

Horiuchi et al. (2008)^38^ recommended to aggregate decomposition results for relatively short time intervals rather than to carry out one decomposition for the entire period of data. For example, in our analyses, we decompose some longevity changes between 1960 and 1973 within annual intervals. To improve our estimates, we decompose the change in each year between 1960 and 1973 by assuming proportional changes during the single-year period and then aggregate the 13 sets of decomposition results, instead of decomposing the change in the entire 13-year period directly by assuming proportional changes from 1960 to 1973. The division of the four periods is motivated by the choice of having time windows of equal width, giving equal importance to each period. In this way, we avoid giving more weight to some periods and ease the interpretability of the analysis. We decompose changes in life expectancy and lifespan inequality by single age, period, and cause of death.

## Supplementary Information

Supplementary Figures.

## Data Availability

The replication scripts, written in the R statistical programming language (R. Core Team, 2013); and the dataset analyzed during the current study, referred to the World Health Organization (WHO) mortality data, are available on the Open Science Framework (OSF) at https://osf.io/yr92s/?view_only=a899e63ae7274374b23aecd457edf425.
