# Supplementary material for: Evaluation of age-specific causes of death in the context of the Italian longevity transition
Source: Sci Rep. 2022 Dec 31;12:22624. doi: 10.1038/s41598-022-26907-3 (PMC9805442; doi:10.1038/s41598-022-26907-3)
Supplement: Supplementary file 1 — Supplementary Figures. [file 41598_2022_26907_MOESM1_ESM.pdf]

Appendix: Evaluation of age-specific causes of death in the context of the Italian longevity transitions

Andrea Nigri[1,*], José Manuel Aburto[2,3,4], Ugofilippo Basellini[5,6], and Marco Bonetti[1,7]

[1]Department of Social and Political Sciences, Bocconi University, Milan, Italy
[2]Leverhulme Centre for Demographic Science and Nuffield College at University of Oxford, UK
[3]Interdisciplinary Centre on Population Dynamics, University of Southern Denmark
[4]Department of Population Health, London School of Hygiene and Tropical Medicine.
[5]Max Planck Institute for Demographic Research (MPIDR), Rostock, Germany
[6]Institut national d'études démographiques (INED), Aubervilliers, France
[7]Carlo F. Dondena Research Centre, Bocconi University, Milan, Italy
[*]andrea.nigri@unibocconi.it

July 28, 2022

A Appendix

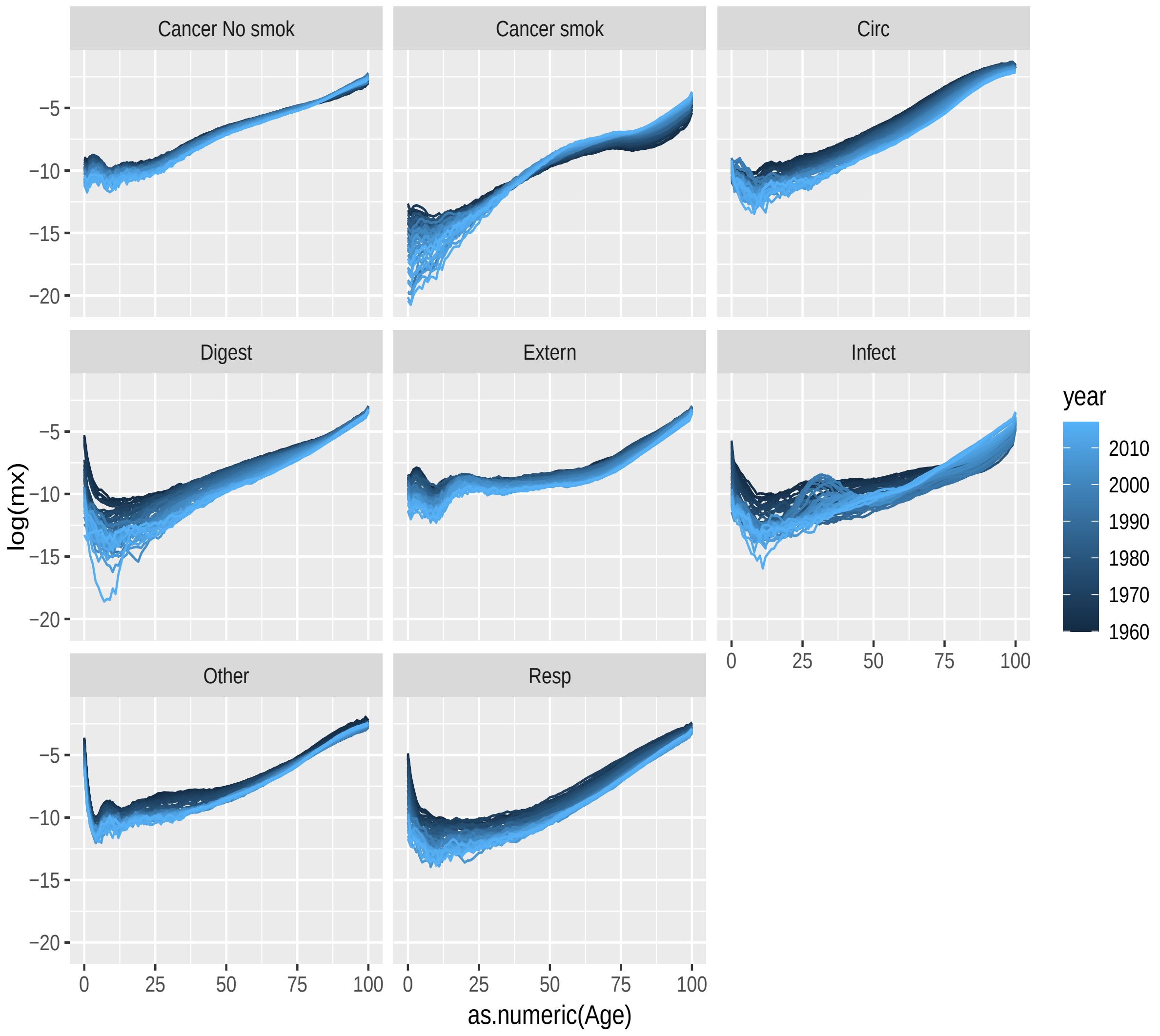

Figure A.1: Female population. Age and cause-specific death rates in log scale. Estimation using PCLM and our ICD classification.

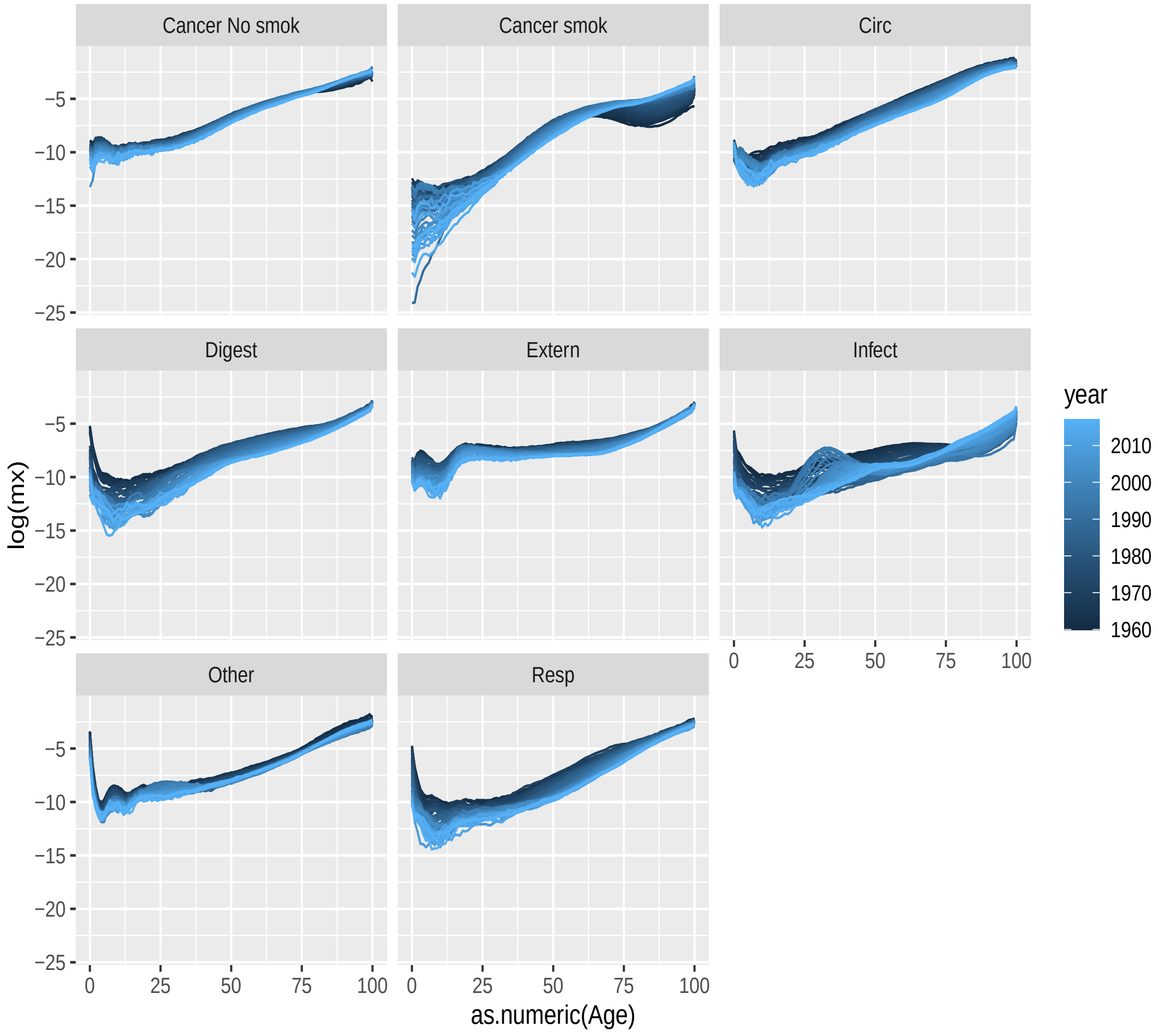

Figure A.2: Male population. Age and cause-specific death rates in log scale. Estimation using PCLM and our ICD classification.

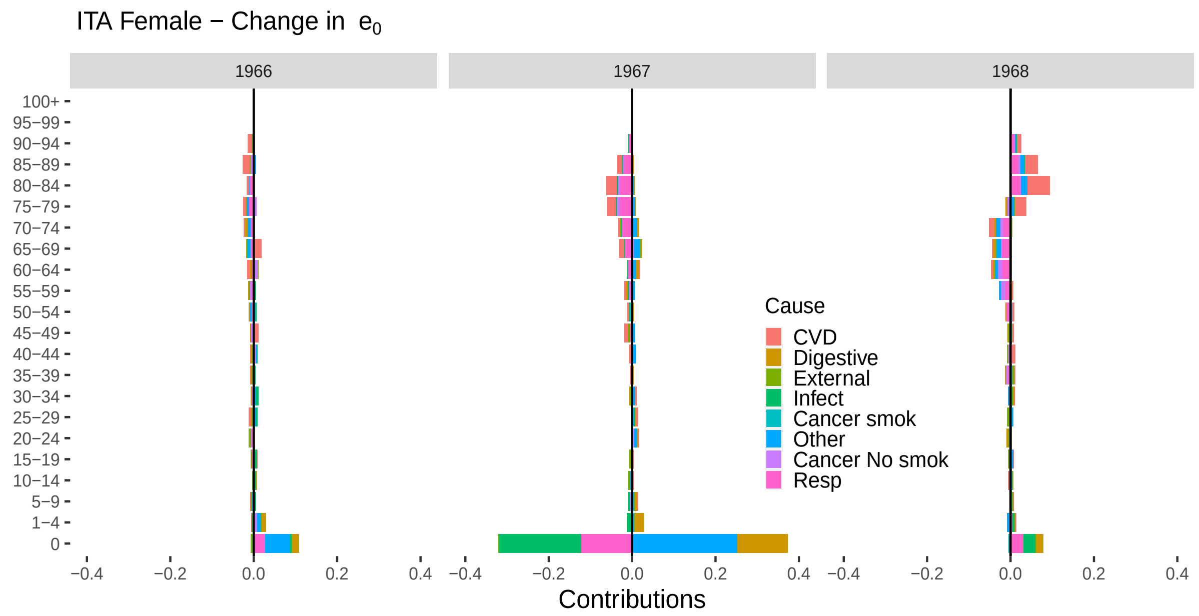

Figure A.3: Yearly contributions to changes in life expectancy for the years 1966–1968, Italian females.

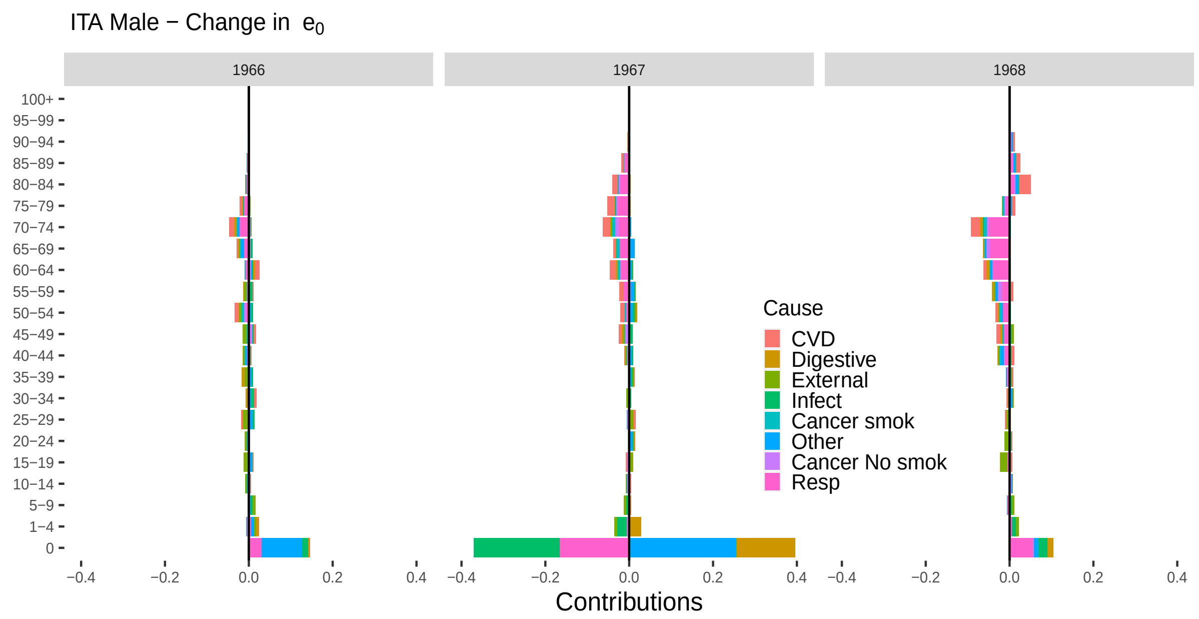

Figure A.4: Yearly contributions to changes in life expectancy for the years 1966–1968, Italian males.
